# Unexpected sensitivity of the highly invasive spider *Mermessus trilobatus* to soil disturbance in grasslands

**DOI:** 10.1007/s10530-020-02348-9

**Published:** 2020-09-07

**Authors:** Nijat Narimanov, Anne Kempel, Mark van Kleunen, Martin H. Entling

**Affiliations:** 1 iES Landau, Institute for Environmental Sciences, Department of Ecosystem Analysis, University of Koblenz-Landau, 76829 Landau, Germany; 2 Institute of Plant Sciences, Department of Community Ecology, University of Bern, 3013 Bern, Switzerland; 3 Ecology, Department of Biology, University of Konstanz, 78457 Konstanz, Germany; 4 Zhejiang Provincial Key Laboratory of Plant Evolutionary Ecology and Conservation, Taizhou University, Taizhou, 318000 China; 5 Institute of Ecology and Evolution, Community Ecology, University of Bern, 3012 Bern, Switzerland

**Keywords:** Araneae, Disturbance, Habitat preference, Invasibility, Linyphiidae, *Oedothorax apicatus*

## Abstract

The dwarf spider *Mermessus trilobatus* (Araneae: Linyphiidae), native to North America, has expanded its range over large parts of Europe within less than fifty years. It is notable for occurring in a wide range of mostly agricultural habitats, while most other invasive spiders in Europe are associated with human buildings. As in other invasive invertebrates and plants, the tremendous colonisation success of *Mermessus trilobatus* might be related to anthropogenic habitat disturbance. Here we aim to test if the invasion success of *Mermessus trilobatus* in Europe is associated with high tolerance towards soil disturbance. We sampled spiders from eight grasslands experimentally disturbed with superficial soil tillage and eight undisturbed grasslands without tillage. Opposite to our expectation, *Mermessus trilobatus* densities decrease sharply with soil disturbance. This is in contrast to several native species such as *Oedothorax apicatus*, which becomes more abundant in the fields after superficial soil tillage. Our study suggests that invasion success of *Mermessus trilobatus* is not connected to a ruderal strategy. The ecological and evolutionary processes behind colonisation success of *Mermessus trilobatus* need to be further investigated.

**Electronic supplementary material:**

The online version of this article (10.1007/s10530-020-02348-9) contains supplementary material, which is available to authorized users.

## Introduction

Despite their essential role in ecosystems (Michalko et al. 2019; Nyffeler and Birkhofer 2017), invasions by spiders have only recently started to receive scientific attention (Nentwig 2015). One of the most widespread alien spider species in Europe is the North American dwarf spider *Mermessus trilobatus* (Araneae: Linyphiidae), formerly known as *Eperigone trilobata* (Millidge 1987; Nentwig 2015; Nentwig and Kobelt 2010; Schmidt et al. 2008). It was first detected in Europe in the late 1970s in the Upper Rhine valley near Karlsruhe in South-West Germany (Dumpert and Platen 1985). The species has undergone a largely concentric range expansion and has been recorded in numerous other countries since 1990, such as Austria, Belgium, Croatia, Czech Republic, France, Great Britain, Hungary, Italy, the Netherlands, Poland, Slovakia, Slovenia, Switzerland, and Ukraine (Hirna 2017). To our knowledge, this rapid spread makes *M. trilobatus* currently the most invasive (sensu Richardson et al. 2000) spider in Europe.

*Mermessus trilobatus* has mostly been collected in open habitats within agricultural landscapes and can be among the most abundant spider species there (Schmidt et al. 2008). Its occurrence in agricultural lands suggests that the invasion success of *M. trilobatus* could be based on a ruderal strategy, whereby it would benefit from reduced competition with native species in disturbed habitats (Elton 1958). Lab experiments confirm that *M. trilobatus* is a poor competitor due to its slightly smaller body size compared to native spiders living in the same habitats (Eichenberger et al. 2009). Furthermore, *M. trilobatus* might benefit from post-disturbance resource influxes to the habitat (e.g. from decomposing plant material), or from altered structure and habitat opening (Lear et al. 2020).

Here we aim to test if *Mermessus trilobatus* benefits from soil disturbance in one of its preferred habitats, perennial hay meadows. We compare its abundance to native linyphiid spiders in replicated experimentally disturbed and control grassland sites, expecting that *M. trilobatus* abundances increase after disturbance.

## Methods

### Field characteristics and sampling

The experiment was conducted in 16 permanent hay meadows in the Canton of Bern, Switzerland, in 2008 (Table S2 in supplementary material). All grassland sites belonged to the same community type and were situated 0.5–50 km from each other. The treatments were randomly assigned to the 16 grassland sites. In each grassland, one plot of 240 m^2^ was used. Eight plots were superficially tilled with a rotary tiller (Figure S1 and Figure S2 in supplementary material) in the first half of April, creating soil and ground surface disturbance (disturbed fields). The vegetation was left to decay. The other eight grasslands served as a control and were mown instead of tilled also in the first half of April, and the mown grass was left to decay (undisturbed fields in the following). Disturbance with the rotary tiller had profound effects, killing part of the vegetation and loosening the soil surface, but still leaving sufficient perennial plants alive for continuous vegetation cover. By contrast, mowing only shortened the vegetation at an early growing stage, which is common practice in this grassland type and was required for a plant introduction experiment reported elsewhere (Kempel et al. 2013), but did not affect the ground surface. The sites received the same set of plant species with variable propagule pressure at the beginning of May for the plant introduction experiment. Most adults of *M. trilobatus* are found in summer (Arachnologische Gesellschaft 2020). Thus, the spiders were sampled in late June to early July, 1–2 months after the disturbance event, which meant that the immediate impact was over, but that the vegetation was still different between disturbed and undisturbed sites. The sown plants were hardly visible at the time of sampling and were therefore unlikely to have affected the spiders in the field. We sampled spiders with a vacuum sampler with an 11 cm diameter nozzle (modified STIHL SH85 blower; Stihl, Waiblingen, Germany). It was lowered 150 times per meadow, each time over a different location, resulting in a sampled area of 1.4 m^2^ per meadow, except for two undisturbed plots with 200 times each, or 1.9 m^2^ (Table S2 in supplementary material). Densities per square metre were analysed to account for this difference in sampling effort. By lowering the nozzle until just above the ground, both the vegetation and ground surface was sampled (Sanders and Entling 2011). All samples were transferred in ethanol (70%) for further identification in the lab.

### Study species

All spiders were identified to species level with the aid of a stereomicroscope (Table S1 in supplementary material). Linyphiid species were identified using “The Spiders of Great Britain and Ireland” by Roberts (1987) and “Spiders of Europe” online key (Nentwig et al. 2020). The non-linyphiid spiders were identified with “Collins Field Guide: Spiders of Great Britain and Northern Europe” by Roberts (1995), names following the World Spider Catalog (Nentwig et al. 2020). To reduce the effects of rare species, we used only species present in at least half of the plots in each treatment group (at least 4). We ended up with eight linyphiids: the invasive species *Mermessus trilobatus* and seven native species, namely, *Agyneta rurestris*, *Erigone atra*, *Erigone dentipalpis*, *Oedothorax apicatus*, *Oedothorax fuscus*, *Pelecopsis parallela* and *Tenuiphantes tenuis*. These are all small (< 3 mm) spider species that live among vegetation close to the ground surface. They represent a gradient in hunting strategies, with *A. rurestris*, *M. trilobatus* and *T. tenuis* being obligatory builders of horizontal sheet webs; *E. atra, E. dentipalpis* and *P. parallela* capturing prey both within and outside webs; and *O. apicatus* and *O. fuscus* being free hunters (ME, personal observation; Cordoso et al. 2011).

### Statistical analysis

We calculated the number of individuals per square meter in each field. We modelled the number of individuals per spider species fitting a multivariate generalized linear model (MvGLM) from mvabund package in R 3.6.1 (R Core Team 2019; Wang et al. 2012). We used a negative binomial distribution as the most flexible and appropriate for count data (O’Hara and Kotze 2010). We analysed soil disturbance (disturbed, undisturbed) as a fixed predictor with the “anova.manyglm” function with correction for multiple tests using the “p.uni” function (test =”LR”) with 100,000 permutations.

## Results

**Fig. 1:**
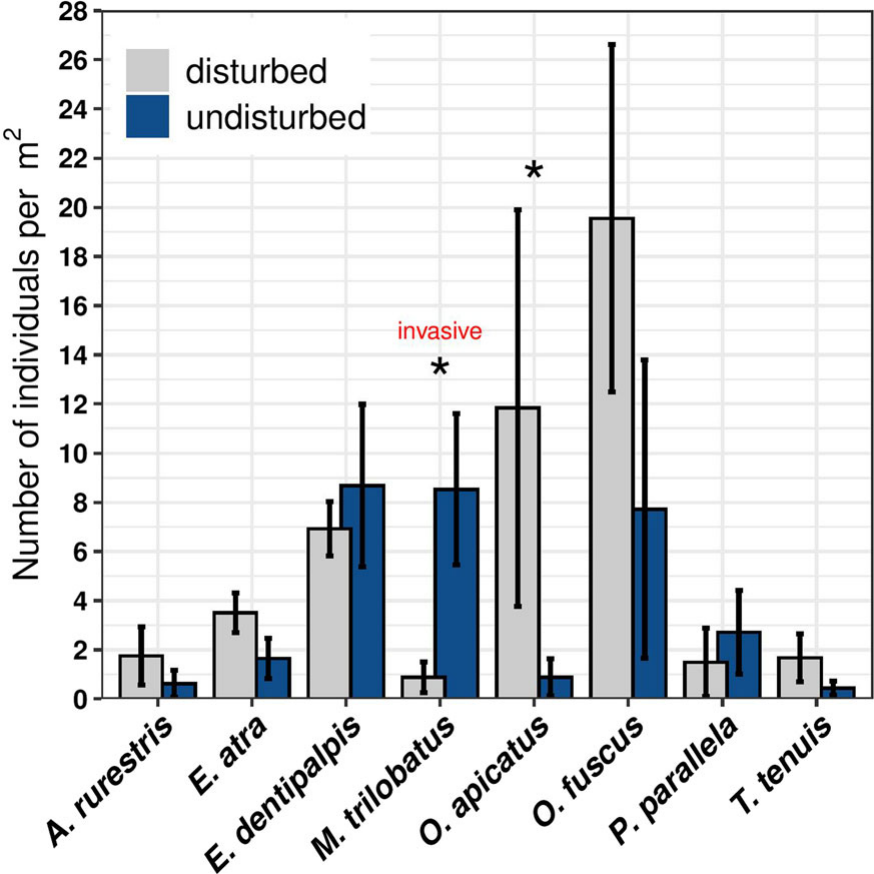
Response of spiders to soil disturbance. The number of individuals per 1 m^2^ for all 8 spider species are illustrated. Spiders were sampled from 8 meadows after soil tillage (disturbed) and 8 meadows without tillage (undisturbed). Mean ± SE are presented, with significant differences marked with asterisk. Invasive species: *Mermessus trilobatus* (Dev = 9.451; *P* = 0.003); Native species: *Agyneta rurestris* (Dev = 0.968; *P* = 0.39), *Erigone atra* (Dev = 2.909; *P* = 0.12), *Erigone dentipalpis* (Dev = 0.283; *P* = 0.61), *Oedothorax apicatus* (Dev = 5.099; *P* = 0.03), *Oedothorax fuscus* (Dev = 1.127; *P* = 0.21), *Pelecopsis parallela* (Dev = 0.194; *P* = 0.64), and *Tenuiphantes tenuis* (Dev = 2.681; *P* = 0.22)

*Mermessus trilobatus* individuals were found in half of the disturbed and in 7 out of 8 undisturbed sites. Community composition of spiders was significantly affected by soil disturbance (Dev = 22.71; *P* = 0.02). Opposite to our expectations, *M. trilobatus* densities were reduced almost 90% after disturbance (Dev = 9.451; *P* = 0.003), and none of the native species showed a comparable decline (Fig. 1). In undisturbed grasslands, *M. trilobatus* was the most abundant spider together with *Erigone dentipalpis*. Densities of *O. apicatus* were approx. 13-fold higher in disturbed than in undisturbed meadows (Dev = 5.099; *P* = 0.03). The other six native linyphiids showed no significant response to the disturbance treatment (Fig. 1).

## Discussion

Opposite to our expectations, our results suggest that the highly invasive spider *M. trilobatus* is more sensitive to soil disturbance than sympatric native European species. One of the native species, *O. apicatus*, even increases in abundance in the disturbed grassland sites. The increase of *O. apicatus* in disturbed grassland does not come as a surprise since they are adapted to live and even overwinter in annual crop fields with little vegetation cover (Mestre et al. 2018; Schmidt and Tscharntke 2005). Furthermore, since mainly cursorial spiders show avoidance behaviour towards intraguild predators like ants (Mestre et al. 2020), *O. apicatus* may benefit from soil disturbance which destroys ant nests. By contrast, the webs of *M. trilobatus* can protect them against predators (Blackledge et al. 2003). *Mermessus trilobatus* uses webs for prey capture (ME, personal observation). The destruction of these webs during disturbance represents a disadvantage. However, native obligatory web builders like *A. rurestris* and *T. tenuis* (ME, personal observation; Cordoso et al. Cardoso et al. 2011) are not sensitive to disturbance, so the hunting mode cannot fully explain the decline of *M. trilobatus*. Thus, other factors such as microclimate, prey availability, or competition with the better disturbance-adapted native species (Eichenberger et al. 2009) are potential mechanisms behind the sensitivity of *M. trilobatus* to disturbance but require further study. From an evolutionary perspective, the reduced adaptation of *M. trilobatus* to soil disturbance compared to European species may be related to the much more recent spread of annual cropping systems in its native North American range, and thus reduced time to co-evolve with intensive land-use.

Irrespective of the mechanisms, the decline of *M. trilobatus* after disturbance raises the question of how it can nevertheless be so successful in European agricultural landscapes. Importantly, the short-term decline of *M. trilobatus* observed here should not be mistaken for a general avoidance of disturbed habitats. Most (86%) of the specimens in Germany have been recorded from grasslands, which depend on regular disturbance of the vegetation layer, i.e. mowing or grazing, in this climatic region. *Mermessus trilobatus* is rarely found both in completely undisturbed habitats such as forests (2.4% of individuals), but also in highly disturbed annual crops (1.3% of individuals) (Arachnologische Gesellschaft 2020). This avoidance of habitats with cultivated soil is in line with the results found in the current experiment.

Possible ecological mechanisms for the success of this species in Europe include the enemy release hypothesis (Roy et al. 2011). Reduced pressure by native predators, parasitoids and pathogens enhances the survival of alien relative to native species. Such potential advantages could be straightforwardly tested experimentally using important enemies of linyphiid spiders such as ants (Hymenoptera: Formicidae) or wolf spiders (Araneae: Lycosidae; Nyffeler 1999). Lastly, it is possible that *M. trilobatus* can spread in its invasive range without being limited by ecological interactions with native species, just as high numbers of native linyphiid spiders are able to coexist in the same habitat.

In summary, our study shows that in contrast to the theory of disturbance-mediated invasion success, *M. trilobatus* does not benefit from soil disturbance. Thus, other potential mechanisms behind its colonisation success remain to be studied, notably its potentially higher reproduction or reduced sensitivity to predators, parasitoids, or pathogens. Given the increasing dominance of invasive spiders in many agricultural (e.g. Hogg et al. 2010) and natural habitats (e.g. Pétillon et al. 2020) across the globe, further studies on their ecology are strongly encouraged.

## Electronic supplementary material

Below is the link to the electronic supplementary material.

Supplementary material 1 (DOCX 1567 kb)

## Data Availability

The dataset generated during and/or analysed during the current study is available in the *Figshare* repository, [10.6084/m9.figshare.12726998.v1]
