# Supplementary material for: Unexpected sensitivity of the highly invasive spider Mermessus trilobatus to soil disturbance in grasslands
Source: Biol Invasions. 2020 Sep 7;23(1):1–6. doi: 10.1007/s10530-020-02348-9 (PMC7801346; doi:10.1007/s10530-020-02348-9)

**Unexpected sensitivity of the highly invasive spider *Mermessus trilobatus* to soil disturbance in grasslands**

*Biological Invasions (Invasion Note)*

Nijat Narimanov*, Anne Kempel, Mark van Kleunen and Martin H. Entling

* Corresponding author:

iES Landau, Institute for Environmental Sciences, Department of Ecosystem Analysis, University of Koblenz-Landau, 76829 Landau, Germany.

E-mail address: narimanov@uni-landau.de / nijat.nariman@gmail.com (Nijat Narimanov).

ORCID identifier: 0000-0003-1321-3243

**Table S1.**

List of spider species found in 16 experimental fields. We identified all adult spiders sampled from 8 disturbed and 6 undisturbed grasslands with 150 sampling pulses and 2 undisturbed fields with 200 sampling pulses per site. Linyphiid species were identified using “The Spiders of Great Britain and Ireland” by Roberts (1987) and “Spiders of Europe” online key (Nentwig et al. 2020). The non-linyphiid spiders were identified with “Collins Field Guide: Spiders of Great Britain and Northern Europe” by Roberts (1995), names following the World Spider Catalog (Nentwig et al. 2020).

| **Family** | **Species** | **Total number of individuals** | |
| --- | --- | --- | --- |
|  |  | **Disturbed**  **grasslands** | **Undisturbed**  **grasslands** |
| Araneidae | *Araniella cucurbitina* (Clerck, 1757) | 2 | 0 |
|  | *Mangora acalypha* (Walckenaer, 1802) | 0 | 3 |
| Gnaphosidae | *Drassyllus praeficus* (L. Koch, 1866) | 0 | 2 |
| Linyphiidae | *Agyneta rurestris* (C. L. Koch, 1836) | 20 | 7 |
|  | Agyneta spp. 1 | 0 | 2 |
|  | Agyneta spp. 2 | 0 | 1 |
|  | *Araeoncus humilis* (Blackwall, 1841) | 7 | 0 |
|  | *Bathyphantes gracilis* (Blackwall, 1841) | 4 | 0 |
|  | *Cnephalocotes obscurus* (Blackwall, 1834) | 0 | 1 |
|  | *Dicymbium nigrum* (Blackwall, 1834) | 3 | 15 |
|  | *Diplostyla concolor* (Wider, 1834) | 1 | 0 |
|  | *Erigone atra* (Blackwall, 1833) | 40 | 19 |
|  | *Erigone dentipalpis* (Wider, 1834) | 79 | 109 |
|  | *Erigonella hiemalis* (Blackwall, 1841) | 0 | 1 |
|  | *Mermessus trilobatus* (Emerton, 1882) | 10 | 104 |
|  | *Micrargus subaequalis* (Westring, 1851) | 0 | 2 |
|  | *Microlinyphia pusilla* (Sundevall, 1830) | 1 | 0 |
|  | *Oedothorax apicatus* (Blackwall, 1850) | 135 | 10 |
|  | *Oedothorax fuscus* (Blackwall, 1834) | 223 | 88 |
|  | *Pelecopsis parallela* (Wider, 1834) | 17 | 31 |
|  | *Tenuiphantes tenuis* (Blackwall, 1852) | 19 | 5 |
|  | *Tiso vagans* (Blackwall, 1834) | 4 | 17 |
| Lycosidae | *Alopecosa pulverulenta* (Clerck, 1757) | 0 | 4 |
|  | *Arctosa leopardus* (Sundevall, 1833) | 7 | 0 |
|  | *Pardosa agrestis* (Westring, 1861) | 2 | 6 |
|  | *Pardosa amentata* (Clerck, 1757) | 2 | 0 |
|  | *Pardosa lugubris* (Walckenaer, 1802) | 0 | 2 |
|  | *Pardosa palustris* (Linnaeus, 1758) | 27 | 20 |
|  | *Pardosa pullata* (Clerck, 1757) | 1 | 6 |
|  | *Piratula latitans* (Blackwall, 1841) | 0 | 19 |
|  | *Trochosa ruricola* (De Geer, 1778) | 1 | 0 |
| Phrurolithidae | *Phrurolitus minimus* (C. L. Koch, 1839) | 0 | 1 |
| Salticidae | *Heliophanus cupreus* (Blackwall, 1841) | 0 | 1 |
|  | *Heliophanus flavipes* (Hahn, 1832) | 1 | 4 |
| Tetragnathidae | *Pachygnatha clercki* (Sundevall, 1823) | 14 | 3 |
|  | *Pachygnatha degeeri* (Sundevall, 1830) | 22 | 48 |
|  | *Pachygnatha listeri* (Sundevall, 1830) | 1 | 0 |
|  | *Tetragnatha extensa* (Linnaeus, 1758) | 2 | 0 |
|  | *Tetragnatha pinicola* (L. Koch, 1870) | 1 | 5 |
| Theridiidae | *Cryptachaea riparia* (Blackwall, 1834) | 0 | 1 |
|  | *Enoplognatha latimana* (Hippa & Oksala, 1982) | 0 | 1 |
|  | *Enoplognatha ovata* (Clerck, 1757) | 0 | 1 |
|  | *Neottiura bimaculata* (Linnaeus, 1767) | 0 | 2 |
|  | *Phylloneta impressa* (L. Koch, 1881) | 12 | 1 |
|  | *Robertus lividus* (Blackwall, 1836) | 0 | 1 |
| Thomisidae | *Xysticus cristatus* (Clerck, 1757) | 0 | 2 |
|  | *Xysticus kochi* (Thorell, 1872) | 1 | 2 |

*Nentwig W, Blick T, Bosmans R, Gloor D, Hänggi A, Kropf C (2020) Spiders of Europe. Online at* [*https://www.araneae.nmbe.ch*](https://www.araneae.nmbe.ch)*, accessed July 2008.* [*https://doi.org/10.24436/1*](https://doi.org/10.24436/1)

*Roberts MJ (1987) The Spiders of Great Britain and Ireland: Linyphiidae and check list. Harley Books, United Kingdom.*

*Roberts MJ (1995) Collins Field Guide: Spiders of Great Britain and Northern Europe. Later prt. edition. ed. HarperCollins, New York.*

**Table S2.**

Experimental grasslands characteristics, locations (Kempel et al. 2013) and sampling effort (number of sampling pulses per site).

| **Site name** | **Soil disturbance** | **Latitude** | **Longitude** | **Sampling effort** |
| --- | --- | --- | --- | --- |
| Heimiswil | disturbed | N47° 03′ 58″ | E7° 39′ 58″ | 150 |
| Signau | undisturbed | N46° 56′ 28″ | E7° 45′ 35″ | 200 |
| Rüderswil | disturbed | N46° 59′ 31.81″ | E7° 42′ 49.31″ | 150 |
| Rüderswil | undisturbed | N46° 59′ 02.51″ | E7° 42′ 59.73″ | 200 |
| Kräiligen | undisturbed | N47° 08′ 30″ | E7° 31′ 20″ | 150 |
| Bätterkinden | disturbed | N47° 07′ 34″ | E7° 32′ 17″ | 150 |
| Büren a. d. Aare | disturbed | N47° 08′ 35″ | E7° 23′ 22″ | 150 |
| Mülchi | undisturbed | N47° 06′ 03″ | E7° 28′ 13″ | 150 |
| Hindelbank | disturbed | N47° 02′ 25″ | E7° 33′ 25″ | 150 |
| Bützberg | disturbed | N47° 12′ 19″ | E7° 43′ 24.41″ | 150 |
| Bützberg | undisturbed | N47° 12′ 44.15″ | E7° 45′ 33.31″ | 150 |
| Walliswil | undisturbed | N47° 14′ 51″ | E7° 49′ 30″ | 150 |
| Worblaufen | undisturbed | N46° 59′ 33.86″ | E7° 28′ 43.73″ | 150 |
| Wiedlisbach | disturbed | N47° 14′ 48″ | E7° 39′ 34″ | 150 |
| Albligen | disturbed | N46° 51′ 16.58″ | E7° 19′ 14.28″ | 150 |
| Heimiswil | undisturbed | N47° 03′ 38″ | E7° 38′ 43″ | 150 |

*Kempel A, Chrobock T, Fischer M, Rohr RP, van Kleunen M (2013) Determinants of plant establishment success in a multispecies introduction experiment with native and alien species. PNAS 110:12727–12732. https://doi.org/10.1073/pnas.1300481110*

**Figure S1.**

Soil and ground surface disturbance created by superficial tillage with the aid of a rotary tiller (on the photo: Anne Kempel).


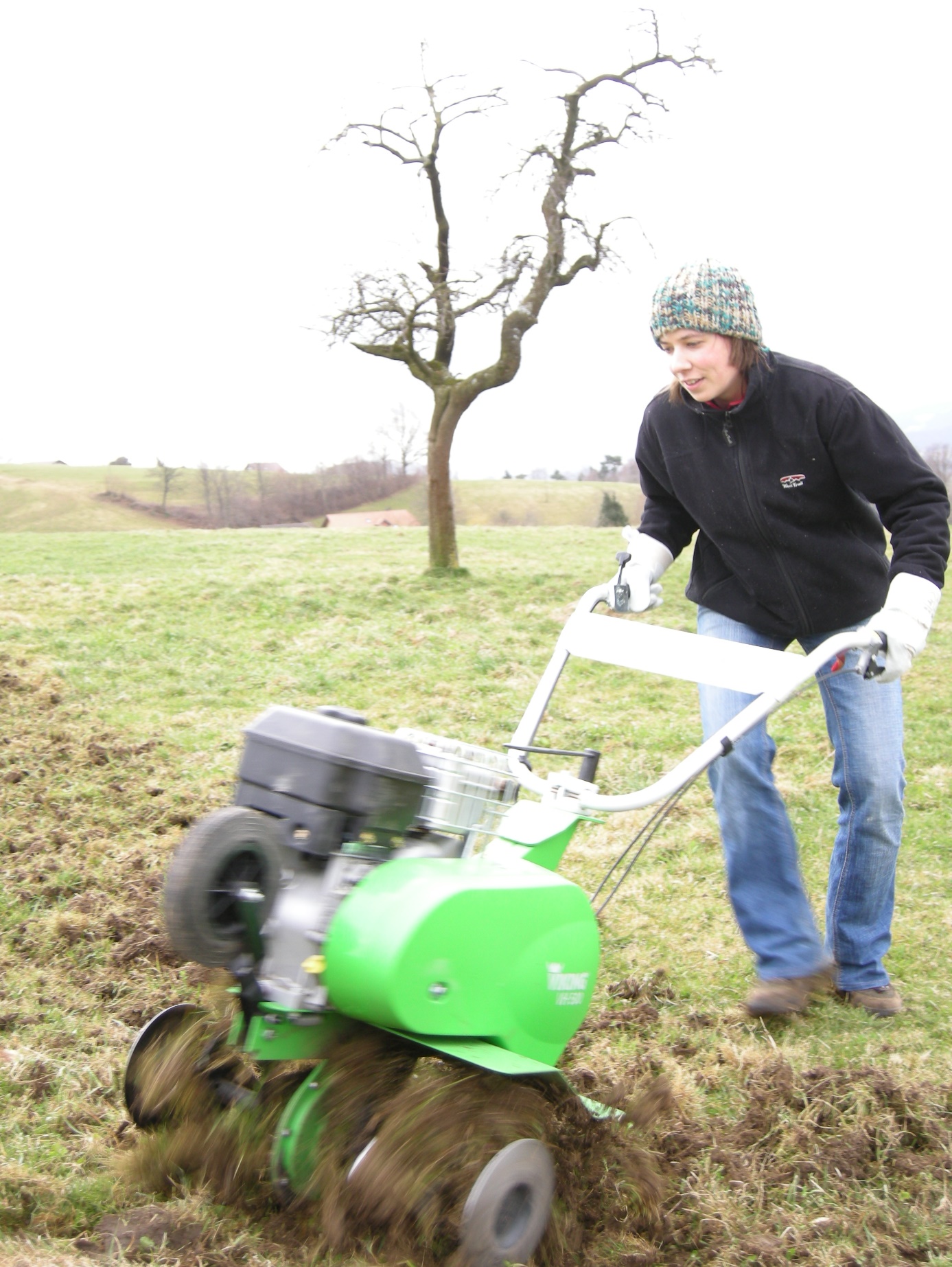


**Figure S2.**

View on a randomly assigned experimental plot of 240 m^2^. The soil and ground surface disturbance was created with a rotary tiller (on the photo: Mark van Kleunen).


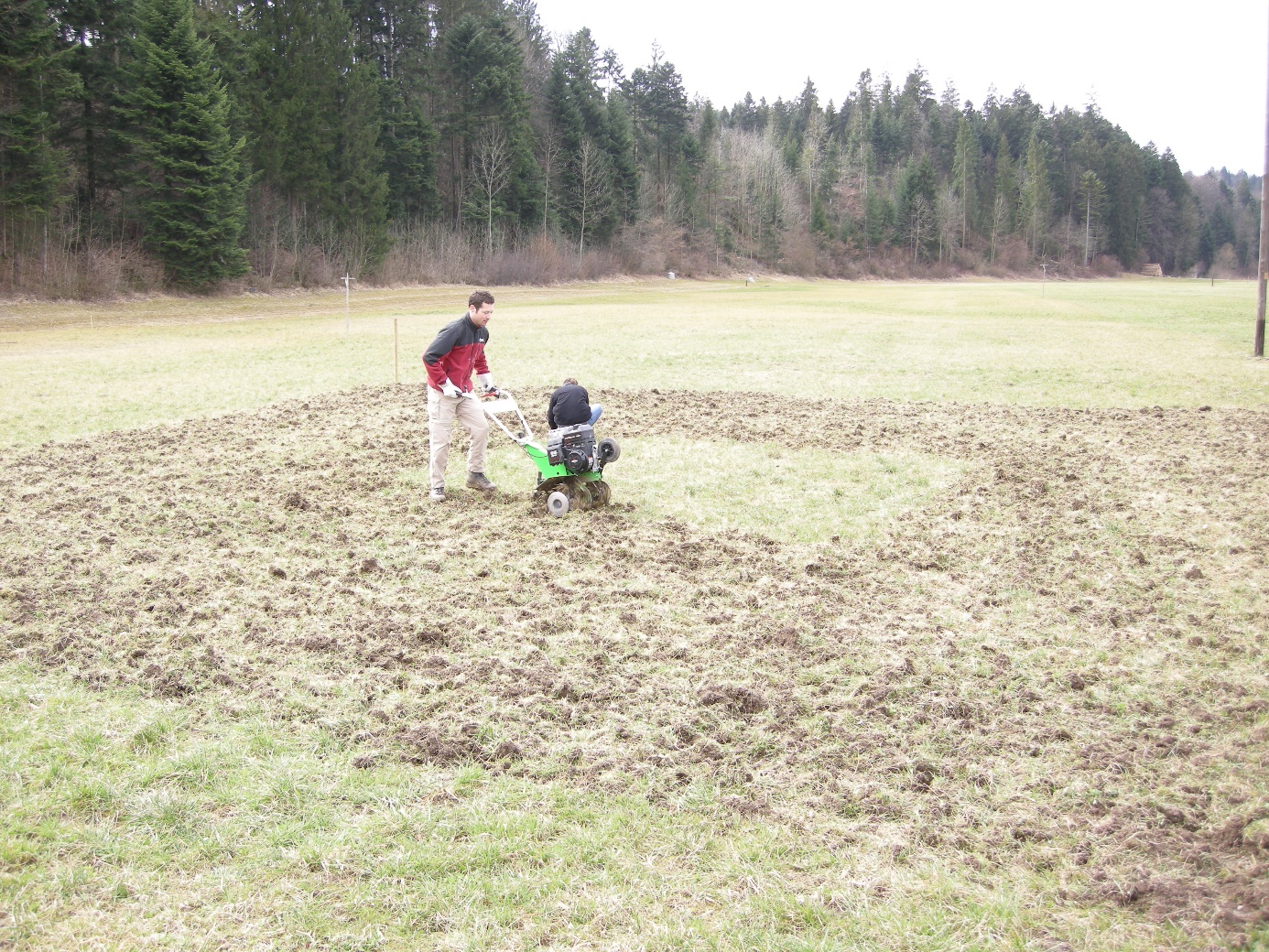

Supplement: Supplementary file 1 — Supplementary material 1 (DOCX 1567 kb) [file 10530_2020_2348_MOESM1_ESM.docx]
